# Insanity

**Published:** 1857-05

**Authors:** 


### INSANITY.

WE do not exactly like to write roughly, but we must inveigh against the mawkish sentimentalities of the times—that *mistaken* philanthropy of a certain class of men, who, brought up in early life without any religious training, yet possessing a high grade of intellect and large-heartedness, strike out into the limitless sea of human amelioration, with heads full of crude theories and hypothetical impossibilities, believing themselves, and causing some of the weaker folk to believe likewise, that they are going to regenerate the world in double quick time—to take a short cut towards the millennial era, and inaugurate a heaven on earth. But having no Bible for their polar star, they soon "fetch up" on the breakers of human depravity, and, in less than a lifetime, a perfect wreck is made of all their hopes, and they conclude to wait for the coming of that more propitious age when men shall be "*less selfish;*" when, if they had read the Bible, they would have found in the outset, that the very first command in the Christian system was directed against that very principle, in the utterance of the Saviour, when he would describe the first step towards an elevation to a better and higher nature: "*Deny thyself, take up thy cross,*" then "*follow me.*"

This very class of persons, Pratt, and Fourier, and Ann Lee, with Dale Owen, and Theodore Parker, and Brisbane, and the lesser lights, such as Pearl Andrews, Hine, Garrison, Thompson, F. Wright, Nichols, and others of a kindred nature, women's rights people, Bloomerites, Radical Abolitionists, phrenologists, vegetarians, water cures, Spiritualists, table tippers, and all that; through all these classes of people there runs a certain vein of *pseudo*-philanthropy and rank infidelity, bordering on Atheism, which shows with perfect plainness that they are radically one and the same thing—enemies of the Christian religion, rushing with reckless indifference into the plausible and untried—all careless of what ruinous consequences may follow, and will follow, should their plans fail. We may with great safety set it down as an incontrovertible fact, that the moment a man begins to improve on Bible philanthropy, that moment he becomes a fool. Turning every woman into a hermaphrodite, the reckless instantaneous freeing of millions of thriftless and improvident slaves, sweeping half the good things of this life from our tables, dismantling our dwellings, cutting the useless buttons from our coats, converting our statuary into lime for manure, covering our cattle with the canvas which records the genius of immortal artists, the dissolution of the marriage tie "made easy" as the unloosing of an old shoe, hugging the heartless murderer to their bosom the moment he is found out, and screening scoundrels of every grade from the penalties of the law, through the tender mercies of the insane asylum—these, we say, are but a part of the attempts to improve on Bible mercy, on Bible polity. How none of them have succeeded, how all of them have miserably failed, and always will fail, present observations teach, and the true historian of future times will have nothing to do but to reiterate the lesson.

It is to one only of these pretended benevolences that we designed to draw attention when we wrote the heading of this article—the plea of insanity, which is now so rife, and which is to become the scapegoat of every infraction of law, and justice, and right. Already has it come to the pass, that if a man eats himself to death, or guzzles bad liquor until he can guzzle no more, or studies himself to a skeleton, and then jumps into the river or puts a bullet through his heart, the merciful verdict is, "*He is insane.*" If he forges his friend's name, or fires his neighbor's dwelling, or his own store, to secure the insurance, or if a young lady allows herself to be abducted by another woman's husband, or a hysterical daughter of a millionaire marries her father's coachman, the convenient cloak of "insanity" is benevolently thrown around these delinquencies and aberrations, and the next day the weak and the unprincipled alike show themselves in the streets, the "observed of all observers"—the lions of the hour.

Is heaven-born charity and her sister, true benevolence, thus to mantle over all that is dishonorable and murderous, and to cover lechery from our sight? These things ought not so to be. The true philanthropist of our day and generation should wake up to the discovery of an effectual remedy for these evils.

But, not to make our article too long, we propose, in short, that all persons be tried for the crimes fairly charged against them. Let the majority of the jury decide on the verdict as to the fact of the act; then let the plea of insanity come in. If not sustained, let the law take its course. If sustained, let the person be committed to an insane asylum for life, if the crime was a capital one, or, if cured of their insanity, to be transferred to the penitentiary for the remnant of their days.

If the act be only a penitentiary offence, let them be sent to the asylum, to remain for life or until cured; and when cured, let them serve the same time in the penitentiary which they would have done had they not been declared insane. For, beyond question, if insane, the asylum is the proper place for them; if not insane, the penitentiary should not be cheated of its workmen. In other words, either have no laws, or enforce those we have enacted.

